# A new mechanism of antibody diversity: formation of the natural antibodies containing LAIR1 and LILRB1 extracellular domains

**DOI:** 10.1093/abt/tbae008

**Published:** 2024-05-21

**Authors:** Yuanzhi Chen, Zhiren Zeng, Ziyou Chen, Na Yuan, Xinya Ye, Chengcheng Zhang, Ningshao Xia, Wenxin Luo

**Affiliations:** State Key Laboratory of Vaccines for Infectious Diseases, Xiang An Biomedicine Laboratory, School of Public Health and School of Life Sciences, Xiamen University, Xiamen 361102, China; National Institute of Diagnostics and Vaccine Development in Infectious Diseases, State Key Laboratory of Molecular Vaccinology and Molecular Diagnostics, National Innovation Platform for Industry-Education Integration in Vaccine Research, Xiamen University, Xiamen 361102, China; State Key Laboratory of Vaccines for Infectious Diseases, Xiang An Biomedicine Laboratory, School of Public Health and School of Life Sciences, Xiamen University, Xiamen 361102, China; National Institute of Diagnostics and Vaccine Development in Infectious Diseases, State Key Laboratory of Molecular Vaccinology and Molecular Diagnostics, National Innovation Platform for Industry-Education Integration in Vaccine Research, Xiamen University, Xiamen 361102, China; State Key Laboratory of Vaccines for Infectious Diseases, Xiang An Biomedicine Laboratory, School of Public Health and School of Life Sciences, Xiamen University, Xiamen 361102, China; National Institute of Diagnostics and Vaccine Development in Infectious Diseases, State Key Laboratory of Molecular Vaccinology and Molecular Diagnostics, National Innovation Platform for Industry-Education Integration in Vaccine Research, Xiamen University, Xiamen 361102, China; State Key Laboratory of Vaccines for Infectious Diseases, Xiang An Biomedicine Laboratory, School of Public Health and School of Life Sciences, Xiamen University, Xiamen 361102, China; National Institute of Diagnostics and Vaccine Development in Infectious Diseases, State Key Laboratory of Molecular Vaccinology and Molecular Diagnostics, National Innovation Platform for Industry-Education Integration in Vaccine Research, Xiamen University, Xiamen 361102, China; State Key Laboratory of Vaccines for Infectious Diseases, Xiang An Biomedicine Laboratory, School of Public Health and School of Life Sciences, Xiamen University, Xiamen 361102, China; National Institute of Diagnostics and Vaccine Development in Infectious Diseases, State Key Laboratory of Molecular Vaccinology and Molecular Diagnostics, National Innovation Platform for Industry-Education Integration in Vaccine Research, Xiamen University, Xiamen 361102, China; Department of Physiology, University of Texas Southwestern Medical Center, Dallas, TX 75390, United States; Department of Developmental Biology, University of Texas Southwestern Medical Center, Dallas, TX 75390, United States; State Key Laboratory of Vaccines for Infectious Diseases, Xiang An Biomedicine Laboratory, School of Public Health and School of Life Sciences, Xiamen University, Xiamen 361102, China; National Institute of Diagnostics and Vaccine Development in Infectious Diseases, State Key Laboratory of Molecular Vaccinology and Molecular Diagnostics, National Innovation Platform for Industry-Education Integration in Vaccine Research, Xiamen University, Xiamen 361102, China; Research Unit of Frontier Technology of Structural Vaccinology, Chinese Academy of Medical Sciences, Xiamen 361102, China; State Key Laboratory of Vaccines for Infectious Diseases, Xiang An Biomedicine Laboratory, School of Public Health and School of Life Sciences, Xiamen University, Xiamen 361102, China; National Institute of Diagnostics and Vaccine Development in Infectious Diseases, State Key Laboratory of Molecular Vaccinology and Molecular Diagnostics, National Innovation Platform for Industry-Education Integration in Vaccine Research, Xiamen University, Xiamen 361102, China

**Keywords:** Plasmodium falciparum, inhibitory immune receptors, antibody rearrangement, receptor-containing antibodies, antibody engineering

## Abstract

The recent discovery of public antibodies targeting *Plasmodium falciparum*-encoded repetitive interspersed families of polypeptides (RIFINs), which contain extracellular immunoglobulin-like domains from LAIR1 or LILRB1, constitutes a significant step forward in comprehending the reactivity of the *Plasmodium* parasite. These antibodies arise from unique B cell clones and demonstrate extensive cross-reactivity through their interaction with *P. falciparum* RIFINs. LAIR1 and LILRBs are specialized type I transmembrane glycoproteins, classified as immune inhibitory receptors, restricted to primates and mainly found on hematopoietic cells. They are instrumental in modulating interactions within the tumor microenvironment and across the immune system, and are increasingly recognized as important in anti-cancer immunotherapy and pathogen defense. The presence of LAIR1/LILRB1-containing antibodies offers new insights into malaria parasite evasion strategies and the immune system’s response. Additionally, the innovative method of integrating extra exons into the antibody switch region is a noteworthy advancement, enriching the strategies for the generation of a varied array of bispecific and multispecific antibodies.

## Teaser

Statement of Significance: Uncovering antibodies with LAIR1/LILRB1 domains provides new insights into malaria immunity, opens up avenues for antibody diversification, and proposes novel approaches to antibody engineering.

## Introduction

The remarkable diversity within antibody repertoires is crucial for the recognition of a broad spectrum of antigens with elevated specificity and affinity. This diversity in antigen-binding sites of antibodies in humans arises from two core mechanisms. First is the unpredictable recombination of gene segments that form the variable regions of immunoglobulin heavy and light chains, known as V(D)J recombination. The second mechanism is the spontaneous modification of amino acids within these variable regions, termed somatic hypermutation (SHM). These processes, which are deeply rooted in evolutionary biology, function at distinct phases of B cell development within different tissue environments. Specifically, V(D)J recombination is primarily active in the bone marrow during the initial stages of B cell maturation, while SHM predominantly occurs in secondary immune organs, often following activation by antigen-specific T helper cells. The synergy of these processes results in a tremendous variety of antigen-binding site configurations [1, 2]. Although V(D)J recombination and SHM are fundamental to adaptive immune responses, acting as the primary strategies to diversify antibody antigen-binding capabilities, their range may be insufficient to cover the infinite theoretical diversity of antigenic molecules found in nature. Hence, exclusive reliance on these traditional diversification strategies may not guarantee complete coverage of the extensive antigenic landscape. Recent discoveries have unveiled additional and updated mechanisms of antibody rearrangement and mutation processes that contribute to unique immunoprotective effects [2–7]. These advancements not only enhance our understanding of B cell humoral immunity but also provide innovative perspectives for the development of therapeutic antibody medications and artificial immune interventions.

*Plasmodium* species, ancient organisms with a history spanning millennia, have intricately coevolved alongside humans. This prolonged association has enabled these malaria-causing parasites to devise sophisticated survival strategies within their human hosts. Central to their survival are the blood stages, where the parasites undergo multiplication within erythrocytes (red blood cells). This phase is crucial for their lifecycle, as it not only facilitates their proliferation but also offers a shield against the host’s immune responses. Once they invade the erythrocytes, *Plasmodium* species initiate a remarkable process of cellular remodeling. They achieve this by exporting an array of proteins to the erythrocyte surface [8]. Following infection of red blood cells by the malignant *Plasmodium falciparum*, a variety of proteins such as *P. falciparum* erythrocyte membrane protein 1 (PfEMP1), the subtelomeric variant open reading frame genes encoding proteins (STEVOR), and *P. falciparum*-encoded repetitive interspersed families of polypeptides (RIFINs) are expressed [9–11]. RIFIN proteins, the most abundant and antigenically diverse molecules in *P. falciparum*, are products of an extensive gene family. This family includes 150–200 rif genes in each parasite’s genome, as detailed by Kyes *et al*. [12]. The RIFIN proteins themselves are characterized by their molecular weight, ranging between 27 and 45 kDa, as identified in 1999 by Kyes *et al*. [13] There are two distinct RIFIN types, A and B, with Type A being more prevalent, accounting for ~70% of the total. A notable difference between these types is an additional 25 amino acids at the N terminus in Type A, absent in Type B, as reported [14, 15]. These proteins are presented on the surface of the infected red blood cells, aiding the parasite in evading the host’s immune surveillance. Meanwhile, these proteins are naturally capable of triggering an immune response. Indeed, in regions heavily affected by malaria, immune responses to RIFINs have been observed, as noted in studies by Abdel-Latif *et al*. in both 2002 and 2003 [16, 17]. Consequently, RIFINs present themselves as potential candidates for developing protective immunity against malaria. Recent studies has identified public anti-RIFIN antibodies that gain *Plasmodium* parasite reactivity through integration of the extracellular immunoglobulin (Ig)-like domains of the leukocyte-associated immunoglobulin-like receptor 1 (LAIR1) [3, 18] or of the leukocyte immunoglobulin-like receptor 1 (LILRB1) [4]. These anti-RIFIN antibodies that contain LAIR1 or LILRB1 fragments are produced by single B cell clones in the donors and cross-react quite broadly. Interestingly, LAIR1, LILRB1, and LILRB2 are receptors of the adhesive polypeptides *P. falciparum*-encoded RIFINs [19, 20].

LILRBs and a relative receptor LAIR1 represent a class of type I transmembrane glycoproteins, classified as immune inhibitory receptors. These receptors are characterized by extracellular Ig-like domains that facilitate ligand binding, and intracellular immunoreceptor tyrosine-based inhibitory motifs (ITIMs), which are adept at attracting phosphatases like SHP1, SHP2, or SHIP. Immunosuppressive myeloid cells in the tumor microenvironment inhibit anti-tumor immunity and support tumor development. LILRBs and the related receptor LAIR1 are immune checkpoint receptors that support the immunosuppressive activity of myeloid cells. Among the proteins that facilitate this immunosuppression, LILRB1 and LAIR1 stand out as two of the key immune checkpoint receptors. LILRB1 and LAIR1 have intracellular ITIMs in their signaling domains, but the individual proteins have different functions. The determinants of the distinct functions of these inhibitory receptors likely rest in their interactions with different ligands and other surface proteins, characteristic signaling domains, and expression dynamics in different cell types regulated by various extrinsic cues and transcription factors. LILRBs and LAIR1 are predominantly expressed on human hematopoietic cells. Their role is crucial in interlinking the functionalities of diverse cell types within the tumor microenvironment and the broader immune system, thereby opening up new avenues not only in anti-cancer immunotherapy but also in combating various pathogens [21–29]. This expanded perspective of their functionality underscores their potential in broader immunotherapeutic applications. These findings expanded our understanding of their functionality emphasizes their potential in a wider array of immunotherapeutic strategies. The presence of LAIR1/LILRB1 in public antibodies opens new perspectives on how the malignant malaria parasite manipulates host’s immune cell functions. This insight is invaluable in deciphering the immune evasion tactics of pathogens and the host’s corresponding immune response mechanisms. Moreover, the innovative strategy of inserting an additional exon into the switch region marks a significant advancement in antibody diversification, similar to exon shuffling. This technique holds promise for generating a diverse range of bispecific and multispecific antibodies, thus expanding the therapeutic potential of antibody drugs.

## LAIR1 and natural LAIR1-containing antibodies

**Figure 1:**
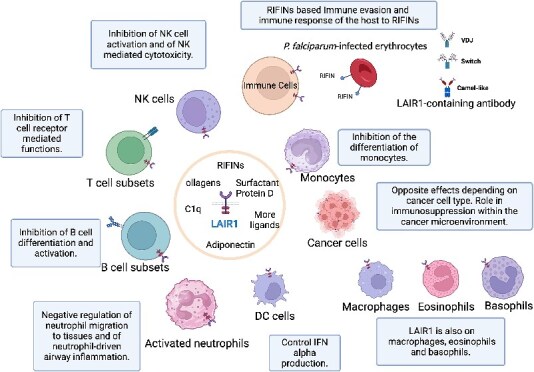
Summary of LAIR1 expression and functions in normal immune cells and tumor cells. This figure was created with BioRender.com.

LAIR1, also recognized as CD305, is a type I transmembrane glycoprotein that consists of a single extracellular Ig-like domain and two intracellular ITIMs [30]. This receptor is broadly expressed across various hematopoietic cells, including monocytes, macrophages, dendritic cells (DCs), eosinophils, basophils, natural killer (NK) cells, and several T and B cell subsets [26, 30–32]. Its extracellular domain interacts with the Gly-Pro-Hyp sequences found in collagen, while its ITIMs facilitate the recruitment of phosphatases SHP-1 and SHP-2. Although LAIR1 serves as an immune inhibitory receptor and seems non-essential for normal hematopoiesis, it plays a significant role in regulating immune system equilibrium and in protecting against tissue damage caused by hyperactive immune responses or autoimmune disorders [21, 23, 25, 26]. LAIR1’s involvement in various cancer types, autoimmune diseases, and infectious diseases has been extensively researched. A range of ligands, including collagens, C1q, MBL, surfactant protein-D (SP-D), RIFINs, and Colec12, have been identified to bind with LAIR1 [19, 33–37]. These interactions underscore LAIR1’s significant role in modulating immune responses across a spectrum of conditions including cancer, autoimmune diseases, and infectious diseases [26, 31, 32], as depicted in Figure 1.

**Figure 2:**
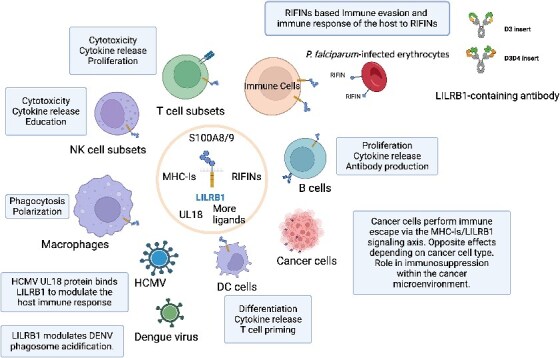
Summary of LILRB1 expression and functions in normal immune cells and tumor cells. This figure was created with BioRender.com.

In individuals exposed to *P. falciparum* in Africa, certain antibodies with LAIR1 insertions demonstrate extensive SHM [3, 18]. This finding, documented by Pieper and Tan *et al*., highlights the unique interaction between these LAIR1 mutations and RIFINs on infected erythrocytes (IEs), setting them apart from the minimal binding affinity of wild-type LAIR1 [3, 19]. These antibodies, featuring LAIR1, arise from various insertion processes, including in the VDJ region, illustrated by antibody MGD21, and the switch region, as seen in antibody MGM5 and MGB47(akin to natural camel-like LAIR1-Fc fusions) [18]. Interestingly, malaria parasites tend to favor B cells with LAIR1 insertions in the VDJ region over the switch region [7, 18]. The LAIR1 segment in these antibodies often harbors amino acid changes in the collagen-binding motifs, enhancing RIFIN binding while reducing collagen affinity. Mutations like T67L, N69S, and A77T in LAIR1 are believed to further strengthen this interaction [18]. Three separate studies delve into the structural basis of how RIFINs bind to LAIR1 and the interplay between LAIR1 antibodies and RIFINs [38–40]. LAIR1 antibodies also work by disrupting the RIFIN–LAIR1 interaction, a testament to the adaptive evolutionary response of these antibodies (Fig. 2). This response provides deeper understanding of their role in immune defense against malaria and possibly other pathogens. MGD21, for instance, has its LAIR1 segment strategically placed at the antigen-binding site, altering the original structure. MGD21’s affinity for RIFINs is notably higher than MGM5’s. Sequence analysis of LAIR1 and these antibodies revealed specific mutations, with MGD21 showing additional variations compared with the germline LAIR1, unlike MGM5 [38, 39]. Furthermore, Xie *et al*. confirmed that LAIR1-binding RIFIN can trigger LAIR1-mediated cell signaling using a nuclear factor of activated T cells-GFP (The green fluorescent protein) reporter system and inhibit wild-type LAIR1 activation by RIFIN molecules, with MGD21 showing greater efficiency in this respect [40]. Finally, the association of LAIR1 was confirmed in 20 out of 47 RIFINs with corresponding signature profiles [39]. This evidence indicates the existence of LAIR1-binding RIFINs in multiple, evolutionarily interrelated *Plasmodium* species, capable of infecting humans, chimpanzees, and gorillas, hinting at a shared evolutionary origin for the human malaria parasite.

## LILRB1 and natural LILRB1-containing antibodies

**Figure 3:**
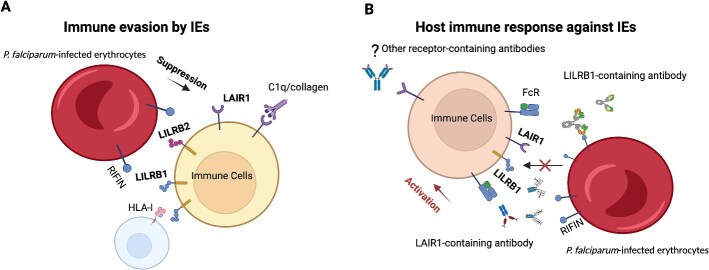
The suppressive effects of RIFINs on the host’s immune defenses and the corresponding immune response of the host to RIFINs. Some RIFINs interact with human inhibitory immune receptors, triggering a dampening signal within the host’s immune system. This interaction, particularly with LAIR1/LILRB1/LILRB2-binding RIFINs, is believed to exacerbate malaria severity (A). In response, the host produces conventional antibodies targeting RIFINs, as well as specialized antibodies incorporating parts of immune receptors. These specialized antibodies are designed to obstruct the RIFIN–receptor interaction, broadly recognize IEs expressing RIFINs that bind to LAIR1 or LILRB1, and aid in eliminating such IEs (B). This figure was created with BioRender.com.

LILRB1, also known as CD85J, ILT2, LIR1, and MIR7, is characterized by four extracellular immunoglobulin domains and four intracellular ITIMs [41, 42]. LILRB1 is the most broadly expressed member of the LILRB family. This receptor is extensively expressed across a range of immune cells, including monocytes, macrophages, DCs, eosinophils, basophils, B cells, and subsets of T cells and NK cells [25, 26]. It is also present in *in vitro* cultured cord blood-derived progenitor mast cells and osteoclasts, making it the most widely expressed member of the LILRB family. LILRB1 interacts with a diverse array of ligands, encompassing both classical (HLA-A, HLA-B, and HLA-C) and non-classical (HLA-E, HLA-F, and HLA-G) major histocompatibility complex class I molecules (MHC-Is), as well as UL18 (a CMV MHC-I homolog), calcium-binding proteins S100A8/9, and RIFINs [19, 43–47]. Notably, antibody-opsonized dengue virus can also engage LILRB1, potentially playing a role in the pathogenesis of dengue infections by dampening immune cell responses [48, 49]. Therefore, LILRB1’s functional spectrum extends beyond the regulation of immune cell activities in relation to MHC-I levels, encompassing potential roles in immune evasion tactics employed by tumors and infectious diseases [23, 25, 26], as depicted in Figure 3.

In malaria-prone regions, antibodies containing LILRB1, similar to those with LAIR1, have been identified in patients. Chen *et al*. conducted a study involving 672 plasma samples from donors in Mali, discovering six individuals with IgG antibodies that included LILRB1 [4]. They isolated B cell clones from three of these individuals, which produced monoclonal antibodies containing LILRB1 that attached to IEs. These B cell clones exhibited substantial DNA insertions in the switch region encoding for non-apical extracellular domains 3 and 4 (D3D4) or just D3 of LILRB1, within the variable-constant (VH-CH1) elbow region. Utilizing mass spectrometry and binding assays, they identified numerous RIFINs that bind to the LILRB1 D3 domain. Structural analysis using crystal and cryo-electron microscopy of a RIFIN complexed with either LILRB1 D3D4 or a D3D4-inclusive antibody Fab revealed a RIFIN–LILRB1 D3 interaction mode resembling the RIFIN–LAIR1 interaction. This Fab displayed a unique triangular structure, with the LILRB1 domains expanding the VH-CH1 elbow but not impacting the VH-VL or CH1-CL pairings. Similar to LAIR1-containing antibodies, B cells isolated from each donor were part of a single clonal family, evidenced by identical inserts and VDJ rearrangements. Although the VH regions showed somatic mutations, the LILRB1 inserts generally did not, except for one antibody with a Y291D mutation. This absence of somatic mutations aligns with the non-self-reactive nature of LILRB1 D3, unlike the collagen-binding LAIR1 domain, which often exhibited mutations to reduce self-reactivity when inserted into antibody genes. However, Chen *et al*.’s study did not identify any antibodies containing LILRB1–D1D2, even though some RIFINs interact with LILRB1–D1D2 as reported [50]. This discrepancy might be due to the lower affinity of the D1D2–RIFIN interaction and the need for somatic mutations to eliminate self-reactivity with β2m, similar to why LAIR1-containing antibodies undergo extensive SHM [3, 18]. Consequently, antibodies containing LAIR1/LIRB1 receptors can trigger specific immune responses against *P. falciparum* without inducing autoimmune reactions (Fig. 2).

## Other findings in genomic inserts contributing to human antibody diversity

Lebedin and colleagues pioneered a method that is independent of specific targets to investigate the existence of insert-containing antibody transcripts among a diverse genetic group [7]. They discovered these inserts in the majority of people studied, with a frequency ranging between 1 in 10 000 and 1 in 1000 000 B cells. Remarkably, ~90% of these inserts were out-of-frame, implying a limited but notable role in the diversity of the B cell repertoire. The ever-changing nature of the antibody repertoire, marked by its spatial and temporal variation, is crucial to acknowledge. The naïve B cell pool in circulation is continually replenished, with estimates suggesting between 1 billion and 100 billion for naïve and total B cells, respectively. Specifically, LAIR1 inserts, absent in those not exposed to malaria, become significant in roughly 5% of malaria-exposed individuals, hinting at the role of inserts in the antibody response. This response might be heightened by pathogen receptors in the inserts or by spontaneous insertions forming new antigen-binding domains, further diversified through somatic mutation. Thus, the frequencies presented in this study should be seen as conservative, possibly underestimating the true impact of inserts on antibody diversity. The study introduces a classification for inserts, splitting them into four types based on origin and insertion sites: nuclear-derived V-D-J inserts (nucVDJ), mitochondrial DNA-derived V-D-J inserts (mtVDJ), and J-CH1 inserts from either nuclear DNA near telomere regions (nucJC) or ERFS (telJC). This categorization highlights the varied origins and formation mechanisms of these inserts. NucVDJ and mtVDJ inserts, generally formed before antigen exposure, originate from repair processes at breaks mediated by the recombination-activating gene (RAG). NucVDJ inserts, often linked with long interspersed nuclear (LINE) elements and large genes, show a predisposition to DNA damage [7]. Conversely, mtVDJ inserts, unique to the mitochondrial chromosome, suggest a different mechanism or state for mitochondrial DNA integration into the nuclear genome. NucJC inserts, primarily in memory B cells, indicate sites broken by activation-induced cytidine deaminase, whereas telJC inserts in naïve B cells imply a role for RAG proteins in their genesis. To understand the general occurrence and frequency of templated DNA sequences in the switch region, Lebedin *et al*. also extracted gDNA from switched memory B cells of European blood donors, amplified these regions, and sequenced them using the Illumina platform [7]. They validated several inserts through biological replicates and deduced a higher frequency of templated inserts, about one in a few hundred switched memory B cells. Conversely, no insert was found in the switch region of naïve B cells. The majority of the inserts originated from genic regions across all chromosomes, notably from genes active in B cells. The genic inserts, constituting 75% of all inserts, came from introns and exons, sometimes including a complete exon with intact splice sites. Interestingly, no insert was detected in T cell receptor or antibody light chain transcripts, suggesting a unique receptiveness of the IGH locus to acquiring inserts [7].

## Concluding remarks

These findings uncover a novel antibody mechanism where antibodies, instead of directly binding to pathogens, recruit a protein to bind an antigen. This mechanism is evident in how *P. falciparum* evades immune responses, utilizing inhibitory receptors LAIR1, LILRB1, and LILRB2 to bind RIFINs, thereby circumventing the typical antibody response [6, 7] (Fig. 2). These receptor-containing antibodies, when secreted, produce a significant amount of proteins in the serum. On one side, they are capable of binding a wide array of RIFINs proteins, significantly broadening the antibody binding spectrum. Concurrently, the high level of secretion of these ligand-binding antibodies allows for competitive binding to RIFINs. This effectively blocks the interaction between RIFINs on the surface of red blood cells and the immune inhibitory receptors LAIR1 and LILRB1 on immune cells, interrupting the immunosuppressive pathway. As a result, the body’s immune response against malaria parasites is bolstered, enhancing the destruction of infected red blood cells and offering a novel avenue for immunological intervention against malaria [50]. It raises the question of whether the human immune system generates other protein-containing antibodies against *P. falciparum*, and whether these proteins also interact with *P. falciparum* RIFINs or other host-expressed proteins. Identifying these antibodies could reveal new receptors or interacting molecules for *P. falciparum* proteins, significantly impacting research on its immune evasion and control strategies. Additionally, unresolved issues include identifying potential RIFIN-binding proteins, the likelihood of similar insertions in other viruses or pathogens, and the feasibility of artificially inserting receptor proteins into antibodies to combat viral immune evasion. The possibility of producing unique, protein-containing antibodies in other chronic human diseases (such as HBV, HCMV, or EBV), autoimmune responses, or tumor microenvironments is also intriguing. Large-scale deep sequencing of antibody in patients could yield fascinating insights [7]. The human antibody heavy chain gene, located on chromosome 14, contrasts with LAIR1 and LILRB1 on chromosome 19 [51]. Exploring this inter-chromosomal antibody rearrangement is crucial for understanding B cell immunity and antibody diversity. Genomic sequencing reveals additional protein or non-coding gene insertions in antibodies, suggesting potential functional roles. These studies note geographical variations in LAIR1/LILRB1-containing antibodies, with lower prevalence in European compared with African malaria patients, indicating a prolonged pathogen–host co-evolution [3, 4, 7, 18]. The selection of LAIR1 and LILRB1 for insertions may be related to their role as RIFIN receptors, their broad expression in immune cells, and compatibility with immunoglobulin-like proteins [19, 26]. At present, we have not identified any publications reporting on natural antibodies containing LILRB2. Given that LILRB2 serves as a receptor for certain RIFINs [20], we anticipate that more further research will be conducted, leading to the discovery of LILRB2-containing antibodies.

These findings also underscore the role of genomic architecture in generating inserts, particularly VDJ and J-CH1 inserts, in protein expression and B cell receptor diversity [7]. This novel class of antibodies involves introducing DNA fragments at double-strand breaks in the antibody’s switch region, akin to exon shuffling [7]. This process holds great potential for generating various bispecific antibodies and is highly valuable for the development of new antibody engineering techniques. Traditional bispecific antibodies couple two antibody recognition fragments into a single molecule, yielding dual recognition and functionality [52, 53]. However, these antibodies often deviate from natural forms, presenting challenges such as complex production processes, instability, and potential immunogenicity [52, 53]. Antibodies containing LAIR1 and LILRB1 inserts represent a class of naturally occurring antibodies in humans, characterized by their broad-spectrum binding to the malignant malaria parasite’s RIFIN proteins. Their more stable structure suggests the possibility of utilizing a similar principle to embed sequences from receptors or interactive proteins that recognize other pathogens into neutralizing antibodies. This approach could produce novel antibodies capable of broad-spectrum recognition and neutralization or elimination of pathogens. Additionally, incorporating active fragments of cytokines into antibodies could generate new types of cytokine-linked antibodies. Lastly, integrating the sequences of nanobodies (VHH) that recognize specific proteins into traditional antibodies could create VHH-containing bispecific antibodies. By concatenating multiple VHH sequences targeting different antigens, it is possible to create multispecific antibodies. These potential modifications, however, require further experimentation and testing, but they hold promise for significant efficacy. Future research should focus on the prevalence of this antibody mechanism and its implications for therapeutic antibody design, while also considering the commonality of mechanisms behind chromosomal translocations and templated insertions.

## Data Availability

Not applicable.
